# The effect of musical sensory orientation training in improving consciousness level in patients with disorders of consciousness: a pilot study

**DOI:** 10.3389/fnins.2025.1610811

**Published:** 2025-07-24

**Authors:** Jiayi Gu, Wei Long, Siqin Zeng, Liang Qin, Yi Dong, Cuini Fang, Xiaoying Zhang

**Affiliations:** 1 Department of Rehabilitation, Hunan Provincial People’s Hospital (The first Affiliated Hospital of Hunan Normal University), Changsha, China; 2 Department of Neurological Rehabilitation, Hunan Rehabilitation Hospital, Changsha, China; 3 School of Rehabilitation Medicine, Capital Medical University, Beijing, China; 4 Music Therapy Center, China Rehabilitation Research Center, Beijing, China

**Keywords:** disorders of consciousness, MSOT, music therapy, CRS-R, pilot study

## Abstract

**Introduction:**

Musical sensory orientation training (MSOT) is an innovative technique to improve the state of consciousness and cognitive function. Compared with traditional arousal therapies, MSOT offers a non-invasive, safe, and easily operable alternative with no side effects. This study aimed to conduct a preliminary investigation into the effect of MSOT in improving consciousness levels in patients with DoC (Disorders of Consciousness), as well as the feasibility of its clinical application, thereby providing reference for future large-sample randomized controlled studies.

**Methods:**

We recruited 42 participants between March 2024 to March 2025, dividing them into two groups: a control group of 21 patients who received conventional treatment for DoC and watched videos of family/friends’ activities and short videos, and an intervention group of 21 patients who received MSOT along with conventional treatment. Patients in both groups were assessed at baseline, week 5 and week 8 of the intervention.

**Results:**

The MSOT group showed significant improvement in Coma Recovery Scale—Revised (CRS-R) subscale and total scores over time (baseline, week 5, week 8). At week 8, the MSOT group demonstrated significantly higher CRS-R scores in communication function and arousal level compared to the control group. Behavioral observations in the MSOT group revealed significantly higher frequencies of name response, sound source tracking, and command-following behaviors.

**Conclusion:**

The results of this study demonstrate that MSOT exhibits certain effect in improving consciousness levels in patients with DoC and demonstrates feasibility in clinical implementation. Extending the intervention duration in future studies may amplify its therapeutic effects.

## Introduction

Disorders of consciousness (DoC) are states of impaired consciousness resulting from serious brain injuries, and includes coma, vegetative state, and minimally conscious state, characterized by reduced levels of arousal and alterations in the content of consciousness (Bender et al., 2023). Patients with DoC face long-term, complex medical and caregiving challenges, presenting one of the most pressing issues in the field of neurorehabilitation. In recent years, several arousal techniques have been developed in the field of DoC rehabilitation, such as pharmacotherapy, hyperbaric oxygen therapy, and brain neuromodulation techniques, which have achieved some success in the treatment of coma (Edlow et al., 2021; Yang et al., 2020). While pharmacologic interventions (e.g., amantadine, zolpidem) have shown promise in enhancing arousal in chronic DoC, their utility is limited by tachyphylaxis, adverse effects (e.g., agitation, insomnia), and variable efficacy across injury endotypes (Giacino et al., 2018; Edlow et al., 2021). Placebo-controlled trials in this vulnerable population also raise ethical concerns, as withholding potential therapies may delay recovery (Fins, 2015). Furthermore, heterogeneity in structural and functional connectivity among DoC patients leads to inconsistent treatment responses, underscoring the need for personalized, non-invasive approaches (Edlow et al., 2021; Thibaut et al., 2019). Moreover, there is insufficient evidence-based support for their use in the arousal treatment of some patients with chronic DoC, and most of the treatments have certain risks (Yao et al., 2023).

With the advancement of neuroscience, neurologic music therapy has been established as a distinctive treatment for DoC. Music, as a sensory stimulus transmitted through auditory receptors, exerts varying effects on patients’ attention and auditory perception depending on its style, tonality, and tempo (Zhang et al., 2021; Liu et al., 2022; O’Kelly et al., 2013). Musical sensory orientation training (MSOT) is an emerging technique of neurologic music therapy that aims to improve the state of consciousness and cognitive function. In recent years, MSOT has gradually been implemented in clinical practice, and research into its mechanisms of action has commenced (Xiao et al., 2023). MSOT utilizes music as a medium to stimulate the sensory systems, thereby facilitating the rehabilitation of patients with DoC. Its fundamental principle is to stimulate the auditory, visual, tactile and motor sensory systems of patients through the rhythm, melody, pitch, and timbre of music, stimulating their sensory perception and motor function (Thaut and Hoemberg, 2014). Compared with traditional arousal therapies, MSOT is non-invasive, safe, and easy to operate, with no side effects. The technique leverages recorded music or live music performances to improve attention retention, enhance the execution of simple commands, and improve cognitive function by engaging patients in simple musical activities, either passively or actively. Various elements of music activate cognitive functions such as working memory, episodic memory, language processing, mental imagery, and attention (Boltzmann et al., 2021). Music therapy interventions employ live music, where therapists improvise on the keyboard with varying rhythms, melodies, and sound effects, eliciting emotional fluctuations of tension, anticipation, and humor (Lichtensztejn et al., 2014). Therapists incorporate various musical elements into patients’ responses, creating opportunities for meaningful interactions.

The study is significant in several areas. Firstly, in the fields of neurology and rehabilitation medicine, DoC is a major challenge and quite difficult to manage. Patients with DoC typically exhibit a range of cognitive, sensory, and motor impairments that severely affect their physical functioning and quality of life (Bender et al., 2023). Effective interventions are crucial to improving the level of consciousness of the patients. Secondly, MSOT is a promising approach that shows potential in improving the prognosis of patients with DoC (Thaut and Hoemberg, 2014; Boltzmann et al., 2021; Lichtensztejn et al., 2014). Further exploration of the efficacy of MSOT could contribute to the development of evidence-based medical interventions. Thirdly, the implementation of MSOT currently lacks standardized guidelines and protocols. Our study integrated auditory, visual, and sensory stimuli through the use of musical elements. During the treatment, the assessment method of the music therapy for DoC was adopted, allowing for concurrent treatment and evaluation. By combining the listening of preferred music with live improvised music interventions, the specific steps of the MSOT were formulated, thereby concretizing the MSOT approach. Therefore, this pilot study prospectively evaluated the therapeutic efficacy of MSOT for consciousness enhancement in DoC, while systematically assessing its clinical implementation feasibility, thereby establishing an empirical foundation for subsequent phase III randomized controlled trials.

## Materials and methods

### Individuals

Given the absence of prior research implementing MSOT in patients with DoC, the expected effect size remains uncertain. We conservatively assume a medium-to-large effect size range (Cohen’s *d* = 0.5–0.8). For the generalized estimating equation (GEE) analysis [α = 0.05; power (1-β) = 0.80], with intervention condition (MSOT vs. video-based multisensory stimulation) as the between-subject factor and time [week 1 (t0), week 5 (t1), week 8 (t2)] as the within-subject factor, we targeted 80% statistical power (corresponding to effect size *f* = 0.30). Power calculations using G*Power 3 indicated that an effective sample size of *n* = 17 per group would achieve 80% power for an effect size of *d* = 0.65 (within the projected medium-to-large range), yielding a total sample size of *n* = 34. To account for an anticipated 10% attrition rate due to data missingness (e.g., invalid questionnaires or participant dropout), we inflated the per-group sample size to 22, resulting in a final total of *n* = 44. This sample size aligns with comparable randomized controlled trials (e.g., Zhang et al., 2021; *n* = 40) and ensures sufficient power to detect effects on primary outcome measures.

**FIGURE 1:**
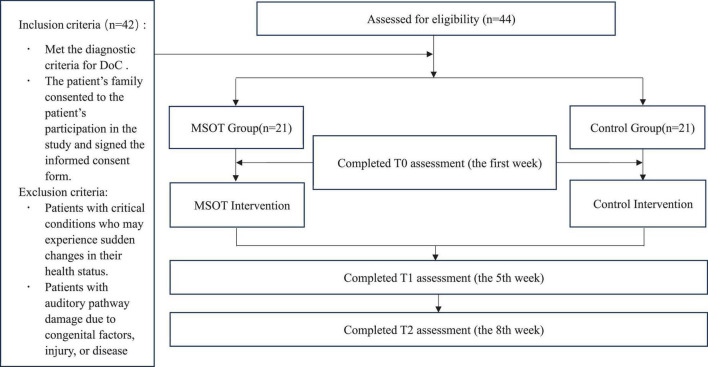
Consort study flowchart.

The study enrolled patients diagnosed with DoC admitted to Hunan Provincial People’s Hospital between March 2024 and March 2025. Convenience sampling initially identified 44 candidates, of whom 42 met the eligibility criteria following the exclusion of two ineligible patients (Figure 1). Inclusion criteria required: (1) met the diagnostic criteria for DoC according to international consensus guidelines; (2) age ≥ 18 years. Exclusion criteria comprised: (1) acute critical illnesses with imminent risk of clinical deterioration; (2) neuroimaging-confirmed structural auditory pathway lesions (congenital malformations, traumatic injuries, or acquired pathologies).

A crucial team member, a doctor of the Department of Rehabilitation Medicine, assisted in presenting the study to potential participants. Posters were displayed in the wards and clinics, detailing the study’s purpose, content, risks and benefits. Written informed consent was obtained from all patients’ legally authorized representatives prior to participation. Participants were allocated to either the intervention (*n* = 21) or control group (*n* = 21).

### Intervention

**FIGURE 2:**
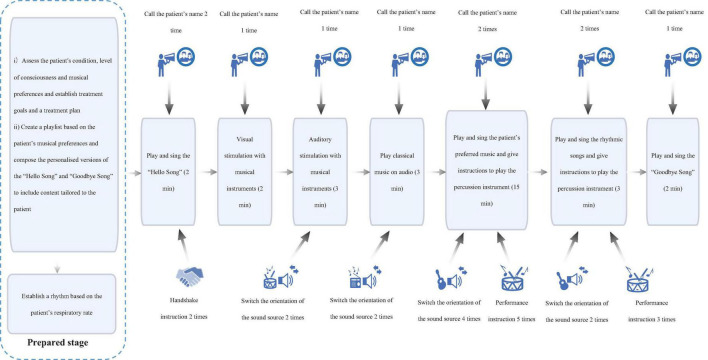
Flowchart of MSOT.

Patients in the intervention group underwent structured MSOT sessions five times weekly, each comprising 30 min of music-mediated multisensory training, over an 8-week period (Figure 2). Specific implementation steps for MSOT showed in Table 1. The MSOT was developed through a systematic synthesis of evidence from prior neurorehabilitation studies (Raglio et al., 2014; Binzer et al., 2016; Magee et al., 2016). The core component of MSOT is the development of an individualized treatment plan based on patient assessment, a dynamic adjustment process through breath synchronization, multi-sensory stimulation (visual tracking/auditory localization), structured musical interactions (customized greetings/farewells tracks), and rhythmic synchronized performance combined with real-time observational feedback. General treatment principles of MSOT showed in Supplemental material 1. All sessions were jointly administered by a certified neurologic music therapist (NMT) and a trained research assistant, with both personnel completing a 20-h competency-based training program in DoC-specific music intervention protocols prior to study initiation.

**TABLE 1:** Specific implementation steps for MSOT.

Items	Contents
Prepared stage 1: Preparedness	✓ The goals and intervention plans for MSOT were determined based on the patient assessment results.
	✓ According to the patient’s preference, 1–2 pieces of classical music, 2–3 preferred songs, and 1–2 rhythmic music tracks were selected.
	✓ Greeting and farewell songs—titled “Hello Song” and “Goodbye Song” respectively—that incorporate personalized patient information, such as their names and ages. Four phrases with repetitive musical and linguistic structures were used to maintain the simplicity of the musical composition, including simple rhythms and recurring melodies. The maximum range of the melody were kept within a single octave. Western tonalities of the same length, such as minor or major keys, were used. The lyrics included “hello”/“goodbye” and the patient’s name.
Prepared stage 2: Establish a breath-synchronized musical rhythm	✓ At the onset of treatment, the patient’s respiratory rate was monitored.
	✓ A note was played each time the patient exhaled: a single low note represented a harmonic progression, such as I-IV-V-I.
	✓ The foundational rhythm and tempo were provided for the opening music, gradually building up to a rhythm based on the patient’s respiratory rate.
Volume modulation response assessment	✓ The Hello Song was played and sung. One verse was sung at a lower volume, while another verse was sung at a higher volume.
	✓ The responses to changes in force and intensity were evaluated.
	✓ The entire verse with the patient’s name was sung at least twice.
	✓ This step was extended if the patient exhibited eye contact, motor responses, or attempts to vocalize.
Visual tracking training	✓ Brightly colored small percussion instruments were positioned in the midline of the patient’s visual field. If the patient turned to one side, the stimulus was positioned in the midline of his/her visual field.
	✓ When the object was fixed, the therapist waited for the patient to “lock” his/her eyes on the target object. Only when the patient’s gaze was fixed on the target, the target was slowly moved from the center of the visual field to the four quadrants around the visual midline: to the left (returning to the center), right (returning to the center), upward (returning to the center), and downward (returning to the center).
	✓ The target object was moved at a speed that the patient could comfortably track. The target object was moved as far as possible within the range of each quadrant that the patient could possibly track.
	✓ In some cases, it was helpful to tell the patient what he or she needed to do: “Look toward the [target object’s name], [patient’s name].”
Auditory localization training	✓ Using a melodic instrument, three to four notes with significant contrast in pitch were played. Pitches used in different registers (for example, C in the three-line octave and F# in the six-line octave) formed dissonant intervals, such as C and F#).
	✓ If the patient’s preference for musical instruments was unknown, a high-pitched handbell was used.
	✓ Auditory stimuli were presented where the patient could not see the stimuli. The musical sound was presented to the patient alone for up to 10 s in the auditory mode.
	✓ In some cases, it was helpful to tell the patient what he or she needed to do, for example, “[patient’s name], turn toward the direction of the sound.” The stimuli were presented outside the patient’s field of vision, but within the range where the patient could demonstrate physical intent, for example, to turn toward the direction of the sound.
	✓ Two musical instruments with very different sounds, such as one with and one without high pitches were played, while giving verbal instructions to turn toward the direction of a specific sound, for instance, “I am going to play two sounds for you. Please turn toward the direction of the guitar sound.”
Multimodal tracking evaluation	✓ Classical music was played, while the patient’s tracking of the directions of visual and auditory stimuli was observed and recorded.
Rhythmic synchronization exercise	✓ A simple percussion instrument was placed in the patient’s hand, and the patient was given performance instructions while listening to his/her preferred music.
	✓ The assistant provided support as needed to help patients play the percussion instrument in time with the music.
	✓ The patient’s responses to performance instructions, as well as to changes in the melody, rhythm, and pitch of the music were observed and recorded.
Continuous rhythm response task	✓ Rhythmic songs were played or sung with performance instructions being continuously given to the patient.
	✓ If needed, the assistant might help the patient touch the instruments.
	✓ The patient’s responses to performance instructions, as well as to changes in the melody, rhythm, and pitch of the music were observed and recorded.
Structured Closure Implementation	✓ The Goodbye Song was played and sung, and the patient’s responses were observed.

**FIGURE 3:**
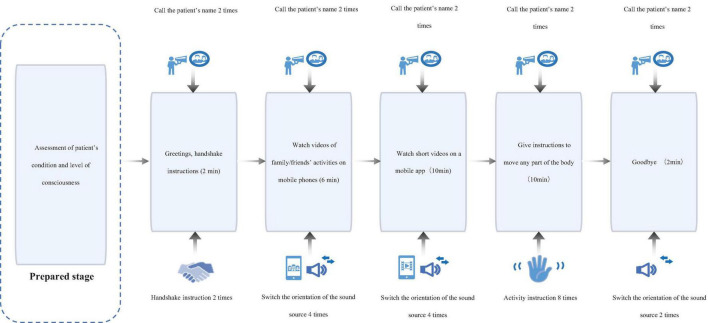
Intervention process for the control group.

In addition to conventional treatment, patients in the control group were exposed to video-based multisensory stimulation consisting of two components: (i) customized audiovisual recordings of family/friends’ daily activities; (ii) passive viewing of neutral short videos via a tablet device (e.g., nature scenes, abstract animations). To standardize tactile input, neutral objects including a plastic comb, textured rubber ball, and silicone massage stick were passively placed in patients’ palms for non-directed tactile exploration during sessions. Interventions were administered in 30-min sessions five times weekly over 8 weeks, with each session jointly supervised by a certified rehabilitation therapist from the research team and the patient’s primary caregiver to ensure protocol adherence (Figure 3).

### Outcome measures

Coma Recovery Scale-Revised (CRS-R) (Giacino et al., 2004): The CRS-R consists of six subscales assessing the auditory function, visual function, motor function, oromotor function, communication, and arousal of the patient. Based on subscale scores, patients were diagnosed with unresponsive wakefulness syndrome, minimally conscious state (MCS), which includes two subcategories known as MCS + and MCS-, or emergence from MCS. To minimize the risk of misdiagnosis, two experienced clinicians conducted the CRS-R assessments twice daily for five consecutive days. The best result from these evaluations was used for the diagnosis (Wannez et al., 2017).

Frequency of behavioral observations: After reviewing the literature (Magee, 2018; Bower et al., 2023), we identified several behavioral parameters for systematic monitoring during therapeutic interventions, including: (a) frequency of onymous response manifestations (head turning, eye blinking, gaze orientation toward auditory stimuli, facial grimacing, and vocalizations upon name recognition); (b) frequency of acoustic source localization through directional gaze orientation; (c) frequency of spontaneous object/instrument manipulation; and (d) frequency of instructional compliance in performing percussive motor sequences. To ensure precise documentation of MSOT-induced responses, all sessions were video-recorded following acquisition of informed consent, with trained evaluators subsequently quantifying response frequencies through frame-by-frame video analysis. According to the calculation, the Cronbach’sα coefficient for the behavioral observation scale was 0.958.

The CRS-R assessments were conducted twice a day for five consecutive days in week 1 (T0), week 5 (T1), and week 8 (T2) of the intervention. The best score from each assessment period was used. Other behavioral assessments were conducted by comparing the cumulative behavioral frequencies at T0, T1, and T2.

### Patient and public involvement statement

This study engaged patients with Disorders of Consciousness (DoC) during the intervention phase. The Musical Sensory Orientation Training (MSOT) protocol was individualized to reflect patients’ musical preferences. Recruitment adhered to strict ethical protocols, with informed consent secured. Throughout the intervention, trained researchers systematically monitored behavioral responses (e.g., name recognition, sound tracking) to refine sensory stimulation in real time. Outcomes were analyzed and communicated within the clinical research team to guide future rehabilitation strategies.

### Statistical analysis

Statistical analyses were performed using SPSS 22.0 for Windows (SPSS, Chicago, IL, United States). The Kolmogorov–Smirnov test was used to test the normality of the data. Normally distributed data were expressed as mean (standard deviation) and non-normally distributed data were expressed as median (interquartile range). The *t*-test and chi-square test were used to assess the balance of the baseline characteristics of the patients between the two groups. A generalized estimating equation model was developed to assess the longitudinal changes in outcome indicators, with outcome indicators at each time point as the dependent variable, and intervention, time, and the interaction between treatment and time as the independent variables. Multiple comparisons were conducted using the Bonferroni method, with statistical significance defined as *p* < 0.05.

## Results

### Participants’ characteristics

A comparative analysis was performed between the two groups regarding age, gender, educational level, disease diagnosis, disease course (defined as days from onset to enrollment), the type of arousal medication used and other arousal-promoting interventions (including duration/frequency of such treatments). No statistically significant differences were observed between the groups (*p* > 0.05; see Table 2).

**TABLE 2:** Comparison of patients’ general information.

Characteristics		Mean ± SD/n		t/χ^2^	*p*
		MSOT group (*n* = 21)	Control group (*n* = 21)		
Age		52.76 ± 18.212	49.86 ± 18.445	0.514	0.610
Course of disease (days)		75.57 ± 82.442	76.52 ± 81.632	−0.038	0.970
Total duration of hyperbaric oxygen therapy (hours)		27.33 ± 1.041	27.07 ± 0.795	0.916	0.365
Frequencies of transcranial magnetic stimulation		41.14 ± 13.774	40.95 ± 13.695	0.045	0.964
Frequencies of direct current stimulation		45.10 ± 2.166	45.19 ± 1.504	−0.166	0.869
Frequencies of acupuncture		36.52 ± 1.861	36.57 ± 1.326	−0.096	0.924
Pharmacological arousal therapy	Amantadine	3	4	1.626	0.654
	Zolpidem	1	1		
	Consciousness-restoring Chinese herbal medicine	5	2		
	None	12	14		
Types of diagnosis	Brain trauma	6	6	0.387	0.943
	Cerebral hemorrhage	11	10		
	Cerebral infarction	1	2		
	Others	3	3		
Gender	Male	14	13	0.104	0.747
	Female	7	8		
Level of education	Primary school	3	2	0.948	0.814
	Middle school	6	7		
	High school or technical secondary school	7	5		
	Junior college and above	5	7		

Table 2 presents the baseline characteristics of the intervention (MSOT) and control groups, demonstrating their comparability at the study outset. Both groups had similar demographic and clinical profiles, with no significant differences in age (52.76 ± 18.21 vs. 49.86 ± 18.45 years, *p* = 0.610), disease duration (75.57 ± 82.44 vs. 76.52 ± 81.63 days, *p* = 0.970), or total hyperbaric oxygen therapy hours (27.33 ± 1.04 vs. 27.07 ± 0.80, *p* = 0.365). Frequencies of adjunct therapies (e.g., transcranial magnetic stimulation, acupuncture) were also balanced (*p* > 0.05). Pharmacological arousal therapies and diagnosis types (e.g., brain trauma, cerebral hemorrhage) were similarly distributed (*p* > 0.05). Gender (male: 14 vs. 13) and education levels (e.g., junior college and above: 5 vs. 7) showed no disparities (*p* > 0.05). This homogeneity supports the validity of subsequent outcome comparisons, as confounding variables were minimized.

### Intervention effects on outcomes

#### Interaction effects of generalized estimating equations

Table 3 presents the results of the generalized estimating equation (GEE) analysis, which evaluates the longitudinal effects of the intervention across multiple timepoints (T0: baseline; T1: mid-intervention; T2: post-intervention). The analysis reveals significant group-by-time interactions (*p* < 0.05) for all assessed Coma Recovery Scale-Revised (CRS-R) subscales (auditory, visual, motor, oromotor, communication, arousal) and total score, as well as all behavioral observation frequencies (responses to name calling, sound source tracking, spontaneous manipulation of instruments or objects, following instructions), at both T1 and T2 follow-ups. The significant interactions indicate that the trajectory of improvement over time differed significantly between the intervention group and the control group, with the intervention group demonstrating substantially greater improvements.

**TABLE 3:** The combined effects of GEE analysis for the comparison of outcome.

Item	Variable	β	95%CI	SE	Wald’sχ^2^	*p*
Scores of CRS-R						
Auditory function						
	Intercept	1.333	1.029, 1.638	0.156	73.500	<0.001*
	Group (exp)^a^	−0.429	−0.871, 0.014	0.226	3.604	0.058
	Time (T2)^b^	0.762	0.391, 1.133	0.189	16.193	<0.001*
	Time (T1)^b^	0.381	0.173, 0.589	0.106	12.923	<0.001*
	Group (exp)*time (T2)^c^	0.857	0.276, 1.438	0.297	8.359	0.004*
	Group (exp)*time (T1)^c^	0.333	0.020, 0.646	0.160	4.360	0.037*
Visual function						
	Intercept	1.667	1.167, 2.166	0.255	42.733	<0.001*
	Group (exp)^a^	−0.619	−1.235, −0.003	0.315	3.874	0.049*
	Time (T2)^b^	0.619	0.235, 1.003	0.196	9.969	0.002*
	Time (T1)^b^	0.333	0.092, 0.574	0.123	7.350	0.007*
	Group (exp)*time (T2)^c^	1.524	0.894, 2.154	0.321	22.494	<0.001*
	Group (exp)*time (T1)^c^	0.476	0.128, 0.824	0.178	7.192	0.007*
Motor function						
	Intercept	2.381	1.829, 2.933	0.282	71.526	<0.001*
	Group (exp)^a^	–0.095	–0.801, 0.611	0.360	0.070	0.792
	Time (T2)^b^	0.619	0.052, 1.186	0.290	4.573	0.032*
	Time (T1)^b^	0.190	–0.061, 0.442	0.128	2.211	0.137
	Group (exp)*time (T2)^c^	1.286	0.490, 2.082	0.406	10.019	0.002*
	Group (exp)*time (T1)^c^	0.619	0.218, 1.020	0.205	9.147	0.002*
Oromotor function						
	Intercept	1.048	0.840, 1.255	0.106	97.731	<0.001*
	Group (exp)^a^	–0.048	–0.356, 0.261	0.158	0.091	0.763
	Time (T2)^b^	0.238	0.056, 0.420	0.093	6.563	0.010*
	Time (T1)^b^	0.095	–0.030, 0.221	0.064	2.211	0.137
	Group (exp)*time (T2)^c^	0.619	0.265, 0.973	0.181	11.752	0.001*
	Group (exp)*time (T1)^c^	0.333	0.087, 0.579	0.126	7.048	0.008*
Communication function						
	Intercept	0^d^	–	0^d^	0.000	1.000
	Group (exp)^a^	0^d^	–	0^d^	0.000	1.000
	Time (T2)^b^	0.095	–0.030, 0.221	0.064	2.211	0.137
	Time (T1)^b^	0^d^	–	0.000	–	<0.001*
	Group (exp)*time (T2)^c^	0.619	0.294, 0.944	0.166	13.972	<0.001*
	Group (exp)*time (T1)^c^	0.286	0.092, 0.479	0.099	8.400	0.004*
Arousal						
	Intercept	1.762	1.537, 1.987	0.115	235.648	<0.001*
	Group (exp)^a^	0.048	–0.233, 0.328	0.143	0.111	0.740
	Time (T2)^b^	0.143	–0.007, 0.293	0.076	3.500	0.061
	Time (T1)^b^	0^d^	–	<0.001	–	<0.001*
	Group (exp)*time (T2)^c^	0.714	0.432, 0.996	0.144	24.609	<0.001*
	Group (exp)*time (T1)^c^	0.381	0.173, 0.589	0.106	12.923	<0.001*
Total scores of CRS-R						
	Intercept	8.190	6.920, 9.461	0.648	159.708	<0.001*
	Group (exp)^a^	−1.095	−2.773, 0.582	0.856	1.638	0.201
	Time (T2)^b^	2.571	1.075, 4.068	0.764	11.340	0.001*
	Time (T1)^b^	1.048	0.408, 1.687	0.326	10.308	0.001*
	Group (exp)*time (T2)^c^	5.476	3.236, 7.716	1.143	22.956	<0.001*
	Group (exp)*time (T1)^c^	2.233	1.346, 3.321	0.504	21.456	<0.001*
Responses to name calling						
	Intercept	4.095	2.272, 5.919	0.931	19.371	<0.001*
	Group (exp)^a^	−1.429	−3.916, 1.059	1.269	1.267	0.260
	Time (T2)^b^	1.905	0.710, 3.099	0.610	9.767	0.002*
	Time (T1)^b^	1.143	0.699, 1.586	0.226	25.519	<0.001*
	Group (exp)*time (T2)^c^	12.810	7.231, 18.388	2.846	20.257	<0.001*
	Group (exp)*time (T1)^c^	5.286	2.468, 8.104	1.438	13.514	<0.001*
Sound source tracking						
	Intercept	4.857	2.559, 7.156	1.173	17.152	<0.001*
	Group (exp)^a^	−0.619	−3.776, 2.538	1.611	0.148	0.701
	Time (T2)^b^	1.238	−0.207, 2.683	0.737	2.819	0.093
	Time (T1)^b^	0.619	−0.121, 1.360	0.378	2.685	0.101
	Group (exp)*time (T2)^c^	13.619	9.041, 18.197	2.336	33.991	<0.001*
	Group (exp)*time (T1)^c^	7.714	4.561, 10.868	1.609	22.988	<0.001*
Spontaneous manipulation of instruments or objects						
	Intercept	3.476	1.933, 5.019	0.787	19.503	<0.001*
	Group (exp)^a^	−0.905	−2.938, 1.128	1.037	0.761	0.383
	Time (T2)^b^	2.714	0.778, 4.650	0.988	7.551	0.006*
	Time (T1)^b^	1.190	0.349, 2.032	0.429	7.693	0.006*
	Group (exp)*time (T2)^c^	11.524	5.414, 17.634	3.118	13.664	<0.001*
	Group (exp)*time (T1)^c^	5.905	2.090, 9.720	1.947	9.201	0.002*
Following instructions						
	Intercept	3.000	1.101, 4.899	0.969	9.587	0.002*
	Group (exp)^a^	−0.810	−3.374, 1.755	1.309	0.383	0.536
	Time (T2)^b^	1.571	0.470, 2.672	0.562	7.826	0.005*
	Time (T1)^b^	0.857	0.293, 1.422	0.288	8.859	0.003*
	Group (exp)*time (T2)^c^	12.333	6.112, 18.554	3.174	15.098	<0.001*
	Group (exp)*time (T1)^c^	8.048	3.604, 12.491	2.267	12.602	<0.001*

CI, confidence interval; EXP, experimental; GEE, generalized estimating equation; SE, standard error; *p < 0.05. ^a^Reference is control group. ^b^Reference is time (T0). ^c^Reference is group (control)*time (T0). ^d^Numerical results near zero (e.g., |value| < 1 × 10^–10^).

For instance, the significant interaction for auditory function at T1 (β = 0.333, *p* = 0.037) indicates that the intervention group achieved a greater mean improvement from baseline than the control group, with an adjusted difference of 0.333 points (95% CI: 0.020–0.646) at T1. Meanwhile, the interaction effect for at T2 was more substantial (β = 0.857, *p* = 0.004), indicating that the intervention group achieved a mean score increase 0.857 points (95% CI: 0.276–1.438) greater than the control group at T2 relative to T0 baselines.

Similarly, significant effects were also found for visual function (T2:β = 1.524, *p* < 0.001; T1:β = 0.476, *p* = 0.007), motor function (T2:β = 1.286, *p* = 0.002; T1:β = 0.619, *p* = 0.002), oromotor function (T2:β = 0.619, *p* = 0.001; T1:β = 0.333, *p* = 0.008), communication function (T2:β = 0.619, *p* < 0.001; T1:β = 0.286, *p* = 0.004), arousal (T2:β = 0.714, *p* < 0.001; T1:β = 0.381, *p* < 0.001), and total CRS-R score (T2:β = 5.476, *p* < 0.001; T1:β = 2.233, *p* < 0.001), as well as frequencies of behavioral observation, including responses to name calling (T2:β = 12.810, *p* < 0.001; T1:β = 5.286, *p* < 0.001), sound source tracking (T2:β = 13.619, *p* < 0.001; T1:β = 7.714, *p* < 0.001), spontaneous manipulation of instruments or objects (T2:β = 11.524, *p* < 0.001; T1:β = 5.905, *p* = 0.002), following instructions (T2:β = 12.333, *p* < 0.001; T1:β = 8.048, *p* < 0.001). These findings collectively suggest that the intervention not only accelerates functional recovery across sensory, motor, and communicative domains but also leads to significantly greater enhancements in complex behavioral responsiveness compared to the control condition, which are critical for patients with disorders of consciousness.

### Simple effects analysis of time

Given the statistically significant group-by-time interaction effect, further analyses examined simple effects. Tables 4, 5 provides a detailed comparison of within-group and between-group changes across the three timepoints. Table 4 presents the results of a simple effects analysis examining within-group changes over time (T0; T1; T2) using Generalized Estimating Equations (GEE). The intervention group demonstrated significantly greater improvements across all outcomes compared to the control group. For example, in auditory function, the intervention group showed a significant increase of 1.62 points from T0 to T2 (95% CI: 1.07–2.17, *p* < 0.001), whereas the control group had a smaller but also significant increase of 0.76 points (95% CI: 0.31–1.22, *p* < 0.001). Similar patterns emerged for visual function, responses to name calling, spontaneous manipulation of objects, and instruction-following (all *p* < 0.05).

**TABLE 4:** Outcome differences between both groups at T0, T1, and T2 according to GEE.

Item	Outcome by group	Control group		Intervention group	
		Mean difference between time ± SD (95%CI)	*p*	Mean difference between time ± SD (95%CI)	*p*
Scores of CRS-R					
Auditory function					
	T2 vs. T1	0.38 ± 0.126 (0.08, 0.68)	0.007*	0.90 ± 0.164 (0.51, 1.30)	<0.001*
	T2 vs. T0	0.76 ± 0.189 (0.31, 1.22)	<0.001*	1.62 ± 0.228 (1.07, 2.17)	<0.001*
	T1 vs. T0	0.38 ± 0.106 (0.13, 0.63)	0.001*	0.71 ± 0.119 (0.43, 1.00)	<0.001*
Visual function					
	T2 vs. T1	0.29 ± 0.099 (0.05, 0.52)	0.011*	1.33 ± 0.182 (0.90, 1.77)	<0.001*
	T2 vs. T0	0.62 ± 0.196 (0.15, 1.09)	0.005*	2.14 ± 0.255 (1.53, 2.75)	<0.001*
	T1 vs. T0	0.33 ± 0.123 (0.04, 0.63)	0.020*	0.81 ± 0.128 (0.50, 1.12)	<0.001*
Motor function					
	T2 vs. T1	0.43 ± 0.197 (−0.04, 0.90)	0.089	1.10 ± 0.201 (0.61, 1.58)	<0.001*
	T2 vs. T0	0.62 ± 0.289 (−0.07, 1.31)	0.097	1.90 ± 0.285 (1.22, 2.59)	<0.001*
	T1 vs. T0	0.19 ± 0.128 (−0.12, 0.50)	0.411	0.81 ± 0.160 (0.43, 1.19)	<0.001*
Oromotor function					
	T2 vs. T1	0.14 ± 0.076 (−0.04, 0.33)	0.184	0.43 ± 0.108 (0.17, 0.69)	<0.001*
	T2 vs. T0	0.24 ± 0.093 (0.02, 0.46)	0.031*	0.86 ± 0.155 (0.49, 1.23)	<0.001*
	T1 vs. T0	0.10 ± 0.064 (−0.06, 0.25)	0.411	0.43 ± 0.108 (0.17, 0.69)	<0.001*
Communication function					
	T2 vs. T1	0.10 ± 0.064 (−0.06, 0.25)	0.411	0.43 ± 0.108 (0.170, 0.69)	<0.001*
	T2 vs. T0	0.10 ± 0.064 (–0.06, 0.25)	0.411	0.71 ± 0.153 (0.349, 1.08)	<0.001*
	T1 vs. T0	0.00 ± 0.000 (0.000, 0.000)	–	0.29 ± 0.099 (0.050, 0.52)	0.011*
Arousal					
	T2 vs. T1	0.14 ± 0.076 (–0.04, 0.33)	0.184	0.48 ± 0.109 (0.22, 0.74)	<0.001*
	T2 vs. T0	0.14 ± 0.076 (–0.04, 0.33)	0.184	0.86 ± 0.122 (0.56, 1.15)	<0.001*
	T1 vs. T0	0.00 ± 0.000 (0.00, 0.00)	–	0.38 ± 0.106 (0.13, 0.63)	0.001*
Total scores of CRS-R					
	T2 vs. T1	1.52 ± 0.489 (0.35, 2.69)	0.005*	4.67 ± 0.629 (3.16, 6.17)	<0.001*
	T2 vs. T0	2.57 ± 0.764 (0.74, 4.40)	0.002*	8.05 ± 0.850 (6.01, 10.08)	<0.001*
	T1 vs. T0	1.05 ± 0.326 (0.27, 1.83)	0.004*	3.38 ± 0.384 (2.46, 4.30)	<0.001*
Frequencies of behavioral observation					
Responses to name calling					
	T2 vs. T1	0.76 ± 0.563 (−0.59, 2.11)	0.528	8.29 ± 1.847 (3.86, 12.71)	<0.001*
	T2 vs. T0	1.90 ± 0.609 (0.45, 3.36)	0.005*	14.71 ± 2.780 (8.06, 21.37)	<0.001*
	T1 vs. T0	1.14 ± 0.226 (0.60, 1.68)	<0.001*	6.43 ± 1.420 (3.03, 9.83)	<0.001*
Sound source tracking					
	T2 vs. T1	0.62 ± 0.488 (−0.55, 1.79)	0.613	6.52 ± 0.954 (4.24, 8.81)	<0.001*
	T2 vs. T0	1.24 ± 0.737 (−0.53, 3.00)	0.279	14.86 ± 2.216 (9.55, 20.16)	<0.001*
	T1 vs. T0	0.62 ± 0.378 (−0.29, 1.52)	0.304	8.33 ± 1.564 (4.59, 12.08)	<0.001*
Spontaneous manipulation of instruments or objects					
	T2 vs. T1	1.52 ± 0.804 (−0.40, 3.45)	0.174	7.14 ± 1.477 (3.61, 10.68)	<0.001*
	T2 vs. T0	2.71 ± 0.988 (0.35, 5.08)	0.018*	14.24 ± 2.957 (7.16, 21.32)	<0.001*
	T1 vs .T0	1.19 ± 0.429 (0.16, 2.22)	0.017*	7.10 ± 1.899 (2.55, 11.64)	0.001*
Following instructions					
	T2 vs T1	0.71 ± 0.486 (−0.45, 1.88)	0.426	5.00 ± 1.236 (2.04, 7.96)	<0.001*
	T2 vs. T0	1.57 ± 0.562 (0.23, 2.92)	0.015*	13.90 ± 3.124 (6.43, 21.38)	<0.001*
	T1 vs. T0	0.86 ± 0.288 (0.17, 1.55)	0.009*	8.90 ± 2.249 (3.52, 14.29)	<0.001*

CI, confidence interval; SD, Standard deviation; *p < 0.0.

**TABLE 5:** Outcome differences between T0, T1 and T2 in two groups according to GEE.

		T0			T1			T2		
Item	Outcome by group	Mean ± SD	Difference between groups (95% CI)	*p*	Mean ± SD	Difference between groups (95% CI)	*p*	Mean ± SD	Difference between groups (95% CI)	*p*
Scores of CRS-R										
Auditory function										
	Intervention group	0.90 ± 0.164	−0.43 ± 0.226 (−1.09, 0.23)	0.865	1.62 ± 0.196	−0.10 ± 0.283 (−0.92, 0.73)	1.000	2.52 ± 0.249	0.43 ± 0.347 (−0.59, 1.45)	1.000
	Control group	1.33 ± 0.156			1.71 ± 0.204			2.10 ± 0.242		
Visual function										
	Intervention group	1.05 ± 0.184	−0.62 ± 0.314 (−1.54, 0.30)	0.735	1.86 ± 0.236	−0.14 ± 0.352 (−1.18, 0.89)	1.000	3.19 ± 0.313	0.90 ± 0.419 (−0.33, 2.14)	0.464
	Control group	1.67 ± 0.255			2.00 ± 0.261			2.29 ± 0.279		
Motor function										
	Intervention group	2.29 ± 0.225	−0.10 ± 0.360 (−1.15, 0.96)	1.000	3.10 ± 0.212	0.52 ± 0.366 (−0.55, 1.60)	1.000	4.19 ± 0.320	1.190 ± 0.502 (−0.28, 2.66)	0.267
	Control group	2.38 ± 0.282			2.57 ± 0.298			3.00 ± 0.387		
Oromotor function										
	Intervention group	1.00 ± 0.117	−0.05 ± 0.158 (−0.51, 0.41)	1.000	1.43 ± 0.108	0.29 ± 0.163 (−0.19, 0.76)	1.000	1.86 ± 0.169	0.57 ± 0.207 (−0.04, 1.18)	0.086
	Control group	1.05 ± 0.106			1.14 ± 0.122			1.29 ± 0.119		
Communication function										
	Intervention group	0.00 ± 0.000	0.00 ± 0.000 (0.00, 0.00)	–	0.29 ± 0.099	0.29 ± 0.099 (0.00, 0.58)	0.056	0.71 ± 0.153	0.62 ± 0.166 (0.13, 1.11)	0.003*
	Control group	0.00 ± 0.000			0.00 ± 0.000			0.10 ± 0.064		
Arousal										
	Intervention group	1.81 ± 0.086	0.05 ± 0.143 (−0.37, 0.47)	1.000	2.19 ± 0.109	0.43 ± 0.158 (−0.04, 0.89)	0.102	2.67 ± 0.123	0.76 ± 0.193 (0.19, 1.33)	0.001*
	Control group	1.76 ± 0.115			1.76 ± 0.115			1.90 ± 0.149		
Total scores of CRS-R										
	Intervention group	7.10 ± 0.559	−1.10 ± 0.856 (−3.61, 1.42)	1.000	10.48 ± 0.761	1.24 ± 1.066 (−1.89, 4.37)	1.000	15.14 ± 1.153	4.38 ± 1.529 (−0.11, 8.87)	0.062
	Control group	8.19 ± 0.648			9.24 ± 0.747			10.76 ± 1.003		
Frequencies of behavioral observation										
Responses to name calling										
	Intervention group	2.67 ± 0.863	−1.43 ± 1.269 (−5.15, 2.30)	1.000	9.10 ± 2.025	3.86 ± 2.263 (−2.78, 10.50)	1.000	17.38 ± 3.272	11.38 ± 3.507 (1.09, 21.67)	0.018*
	Control group	4.10 ± 0.930			5.24 ± 1.010			6.00 ± 1.262		
Sound source tracking										
	Intervention group	4.24 ± 1.104	−0.62 ± 1.611 (−5.35, 4.11)	1.000	12.57 ± 2.431	7.10 ± 2.769 (−1.03, 15.22)	0.156	19.10 ± 2.993	13.00 ± 3.368 (3.11, 22.89)	0.002*
	Control group	4.86 ± 1.173			5.48 ± 1.326			6.10 ± 1.544		
Spontaneous manipulation of instruments or objects										
	Intervention group	2.57 ± 0.675	−0.90 ± 1.037 (−3.95, 2.14)	1.000	9.67 ± 2.328	5.00 ± 2.567 (−2.56, 12.56)	0.784	16.81 ± 3.416	10.62 ± 3.705 (−0.26, 21.49)	0.062
	Control group	3.48 ± 0.787			4.67 ± 1.103			6.19 ± 1.434		
Following instructions										
	Intervention group	2.19 ± 0.880	−0.81 ± 1.309 (−4.65, 3.03)	1.000	11.10 ± 2.761	7.24 ± 3.004 (−1.58, 16.06)	0.240	16.10 ± 3.563	11.52 ± 3.836 (0.26, 22.78)	0.040*
	Control group	3.00 ± 0.969			3.86 ± 1.184			4.57 ± 1.422		

CI, confidence interval; SD, Standard deviation; *p < 0.05.

Critically, motor function improvements were significant in the intervention group (1.90 points, 95% CI: 1.22–2.59, *p* < 0.001) but non-significant in the control group (0.62 points, 95% CI: −0.07–1.31, *p* = 0.097). The same pattern of significant was observed for oromotor function, communication, arousal, and sound source tracking. This divergence confirmed the effectiveness of the intervention. Importantly, the intervention group sustained improvement from T1 (10.48 ± 0.761) to T2 (15.14 ± 1.153), as evidenced by total CRS-R scores (increase of 4.67 points, *p* < 0.001), which far exceeded the concurrent gains in the control group (1.52 points, *p* = 0.005). These results underscore the superiority of the intervention in driving both functional and behavioral recovery, with significant implications for clinical practice.

### Simple effects analysis of group

Intergroup comparisons demonstrated no significant differences in CRS-R subscales (auditory, visual, motor, oromotor), total score, or behavioral frequencies at baseline (T0) and week 5 (T1) (all *p* > 0.05). By week 8 (T2), the MSOT group exhibited significantly greater improvements versus controls in: communication function (mean difference = 0.62, 95% CI: 0.13–1.11; *p* = 0.003), arousal (0.76, 95% CI: 0.19–1.33; *p* = 0.001), responses to name-calling (11.38, 95% CI: 1.09–21.67; *p* = 0.018), sound source tracking (13.00, 95% CI: 3.11–22.89; *p* = 0.002), and following instructions (11.52, 95% CI: 0.26–22.78; *p* = 0.040). Conversely, no significant intervention effects were detected for auditory function, visual function, motor function, oromotor function, spontaneous manipulation of instruments or objects or CRS-R composite scores at any assessment point, including T2 (*p* > 0.05), details in Table 5.

## Discussion

MSOT, as an innovative treatment, is of great significance for the rehabilitation of patients with DoC. The results of this study showed that with the increased duration of MSOT intervention, patients with DoC showed significant improvements in all subscale scores of the CRS-R and the total CRS-R score, as well as in the frequency of behavioral observations. These findings suggest that MSOT is effective in improving the consciousness levels of patients with DoC, which is consistent with the results of previous studies on the effect of live music on improving consciousness in patients with DoC (Liu et al., 2022; O’Kelly et al., 2013; Raglio et al., 2014; Formisano et al., 2001; Binzer et al., 2016). Research suggests that musical stimuli may elicit responses more effectively than verbal stimuli in patients with DoC (Magee et al., 2023). In live improvisational performances, the appropriate adjustment of musical elements such as rhythm, pitch, mode, and volume to support and match some of the behaviors exhibited by patients such as unconscious manipulation of small instruments, blinking, and finger movements significantly encourages patient engagement in musical interaction (O’Connor and Gray, 2022). In addition, the use of the patient’s preferred music can also enhance the expression of residual functions in patients with DoC (Heine et al., 2017). Therefore, in this study, the intervention protocol of MSOT fully considered the selection of musical pieces and the patients’ musical preferences. The patients with DoC were engaged actively or passively through the use of arousal music, live performances of their preferred music, and improvised songs, which effectively improved their behavioral responses.

The results of this study also revealed no significant differences in all outcome measures between the MSOT and control groups at week 5 of the intervention. However, at week 8 of the intervention, the MSOT group showed significant improvements in communication and arousal scores of the CRS-R, as well as in the frequencies of responses to name-calling, sound source tracking, and instruction-following behaviors compared with the control group. These findings indicate that the duration of MSOT intervention affects its efficacy, with improvements becoming more pronounced over time. Furthermore, the MSOT intervention was significantly more effective than the control in improving communication function, arousal level, and behavioral responses in patients. The advantages of music therapy in improving the level of consciousness in patients with DoC lie in its ability to enhance arousal levels and attention, as well as provide opportunities for communicative interaction (Magee et al., 2022). Improvements in name response/sound tracking (behavioral metrics) reflect arousal and attention, which may precede recovery of higher-order functions (e.g., motor execution). CRS-R subscales like “auditory function” require consistent command-following, whereas behavioral metrics capture transient responses. Total CRS-R improvements likely stem from cumulative gains across subscales, even if individual domains (e.g., motor) lacked significance. The findings of this study corroborate this perspective. In the MSOT intervention, improvised music is introduced which contains the name of the patient and has a rhythm based on the patient’s respiratory rate (Magee, 2018). The presentation of preferred music provides a predictable patient behavioral response that can be adapted during live performance to meet individual needs. This approach reduces overstimulation, while maximizing opportunities for musical interaction, thereby reducing the likelihood of understimulation. However, the 8-week MSOT intervention did not show a significant advantage in improving auditory, visual, motor, and oromotor functions in patients with DoC compared with the control group. This lack of significant improvement in these specific domains is likely attributable to the insufficient duration of the intervention, which may not have been long enough to translate gains in arousal and attention into measurable functional recovery in motor and oromotor systems. Research suggests that for patients with motor dysfunction following stroke or traumatic brain injury, an intervention duration exceeding three months is recommended (Daly et al., 2019; Morris et al., 2018; Kwakkel et al., 2023). However, most studies indicate that the training period required for the rehabilitation of cognitive and speech functions might be even longer (Merceur et al., 2024; Axmadjon, 2024). Therefore, based on evidence from comparable rehabilitation paradigms targeting motor and cognitive recovery, future MSOT trials aiming to capture effects on motor, oromotor, and higher-order cognitive functions should consider a minimum intervention duration of 3 months, with potentially longer durations (e.g., ≥ 6 months) required to adequately assess speech and complex cognitive outcomes.

The observed temporal pattern of treatment response—with significant improvements emerging only at week 8 (T2) rather than week 5 (T1)—suggests two potential neurobiological mechanisms. First, MSOT’s multisensory integration may require prolonged exposure to induce structural neuroplasticity in thalamocortical circuits. Animal models demonstrate that combined auditory-somatosensory stimulation over ≥ 4 weeks upregulates synaptic density in the medial geniculate body and primary sensory cortices, particularly after traumatic axonal injury (Fernández-Espejo et al., 2015; Edlow et al., 2021). This aligns with human fMRI studies showing music-enhanced functional connectivity between auditory cortices and prefrontal regions only after 6–8 weeks of intervention in DoC patients (Boltzmann et al., 2021). Second, behavioral conditioning through repetitive musical cues (e.g., pairing patient’s name with specific melodic phrases) may strengthen associative learning in preserved hippocampal-cortical networks. Classical conditioning paradigms in MCS patients require 100–150 trials over 3–4 weeks to establish reliable stimulus-response patterns (Schiff et al., 2009), matching our 40-session protocol. These mechanisms collectively suggest MSOT operates through both bottom-up sensory pathway modulation and top-down cognitive retraining—a dual-process framework supported by recent thalamic deep brain stimulation studies (Chudy et al., 2018).

The quantitative analysis revealed statistically significant improvements in CRS-R following MOST intervention, with 7/21 patients (33.3%) transitioning from MCS-to sMCS, and 5/21 (23.8%) transitioning from VS/MCS- to MCS + neurobehavioral thresholds. Operational viability was confirmed through protocol adherence rates exceeding 90% across all intervention cohorts.

## Conclusion

The results of this study demonstrate that MOST appears effective in improving consciousness levels in patients with DoC and demonstrates feasibility in clinical implementation. Extending the intervention duration in future studies may amplify its therapeutic effects.

### Limitations

This study has several limitations. Firstly, the study was limited by factors such as manpower, environment, and treatment duration, resulting in reliability that is inferior to that of a large-scale randomized controlled trial (RCT). Secondly, the duration of the intervention was constrained exploration of the effects of a longer intervention period. Thirdly, all outcome measures used were subjective assessment indicators, and the results were, to some extent, influenced by subjective factors. Fourthly, and critically in light of the control condition, the use of video-based multisensory stimulation as the control intervention, while a reasonable ethical compromise for this population, is not a fully inert placebo or matched “sham” version of the MSOT. This design choice means it may not adequately control for non-specific therapeutic effects, such as the attention received from researchers, the novelty of the experience, or the general sensory activation provided by the video content. Consequently, the observed differences between groups might partially reflect these non-specific factors rather than solely the specific therapeutic elements of the active MSOT intervention, potentially impacting the internal validity of the findings regarding the specific efficacy of MSOT itself. Future research should incorporate objective outcome measures (e.g., near-infrared spectroscopy, electroencephalogram analysis) and employ more rigorous control conditions, such as carefully matched sham interventions or active control groups controlling for non-specific factors, to further investigate the specific effects and mechanisms of MSOT intervention in larger-scale RCTs.

## Data Availability

The raw data supporting the conclusions of this article will be made available by the authors, without undue reservation.
