# Correction: The effect of musical sensory orientation training in improving consciousness level in patients with disorders of consciousness: a pilot study

**DOI:** 10.3389/fnins.2026.1840348

**Published:** 2026-04-13

**Authors:** Jiayi Gu, Wei Long, Siqin Zeng, Liang Qin, Yi Dong, Cuini Fang, Xiaoying Zhang

**Affiliations:** 1 Department of Rehabilitation, Hunan Provincial People's Hospital (The first Affiliated Hospital of Hunan Normal University), Changsha, China; 2 Department of Neurological Rehabilitation, Hunan Rehabilitation Hospital, Changsha, China; 3 School of Rehabilitation Medicine, Capital Medical University, Beijing, China; 4 Music Therapy Center, China Rehabilitation Research Center, Beijing, China

**Keywords:** disorders of consciousness, MSOT, music therapy, CRS-R, pilot study

In section Materials and Methods, Intervention, paragraph 1, the reference for Magee et al., 2016 was erroneously written as “Magee W. (2018). Music in the diagnosis, treatment and prognosis of people with prolonged disorders of consciousness. *Neuropsychol. Rehabil*. 28, 1331–1339. 10.1080/09602011.2018.1494003”. It should be “Magee, W. L., Siegert, R. J., Taylor, S. M., Daveson, B. A., and Lenton-Smith, G. (2016). Music therapy assessment tool for awareness in disorders of consciousness (MATADOC): reliability and validity of a measure to assess awareness in patients with disorders of consciousness. *J. Music Ther*. 53, 1–26. doi: 10.1093/jmt/thv017”.

In the **Acknowledgments**, the following text was erroneously omitted: “We also sincerely thank Professor Magee for guiding our team before the early stages of this research were carried out.”

The original version of this article has been updated.

