# Supplementary material for: The effect of musical sensory orientation training in improving consciousness level in patients with disorders of consciousness: a pilot study
Source: Front Neurosci. 2025 Jul 24;19:1610811. doi: 10.3389/fnins.2025.1610811 (PMC12330211; doi:10.3389/fnins.2025.1610811)
Supplement: Supplementary file 1 [file Table_1.docx]

**Supplement material 1:**

**General treatment principles of MSOT**

| **Selection of classical music** |
| --- |
| Music was selected from the patients’ preferences that met the following criteria:   1. Clear and simple melodic lines; 2. Tempo in the range of 80–120 beats/min; 3. Volume in the range of 40–60 dB with frequency ranging from 20–20,000 Hz (within the human hearing range, moderate volume, and appropriate frequency); 4. Positive emotional orientation. |
| **Music therapy pieces were selected according to the treatment goal** |
| 1. Enhancing emotional response: choose soothing, lyrical, or cheerful pieces that hold positive significance for the patient; 2. Increasing attention to music: choose familiar pieces for the patient and introduce variations in intensity, rhythm, mode, and timbre during the performance; 3. Improving compliance with musical instructions: choose rhythmic music that requires live performance, adjusting the tempo in real-time according to the patient’s activity speed. |
